# Demonstrating equivalence across magnetoencephalography scanner platforms using neural fingerprinting

**DOI:** 10.1162/IMAG.a.10

**Published:** 2025-05-21

**Authors:** Zoe Tanner, Lukas Rier, Jessikah Fildes, Gonzalo Reina Rivero, Holly Schofield, Christopher Marani, Niall Holmes, Ryan M. Hill, Vishal Shah, Cody Doyle, James Osborne, David Bobela, Matthew J. Brookes, Elena Boto

**Affiliations:** Sir Peter Mansfield Imaging Centre, School of Physics and Astronomy, University of Nottingham, University Park, Nottingham, United Kingdom; Cerca Magnetics Limited, Nottingham, United Kingdom; QuSpin Inc., Louisville, CO, United States

**Keywords:** neural fingerprinting, magnetoencephalography, MEG, optically pumped magnetometer, OPM

## Abstract

Optically pumped magnetometers (OPMs) have emerged as a viable alternative to superconducting quantum interference devices (SQUIDs) to measure the magnetic fields generated by brain activity. Magnetoencephalography (MEG) systems based on OPMs offer potential advantages over conventional systems. However, there remains a pressing need for techniques and studies that demonstrate equivalence between emergent OPM-based instruments and the established conventional MEG standard. Here, we seek to demonstrate similarities between a 192-channel (64-triaxial sensor) OPM-based system and a 275-channel SQUID-based system, when measuring data during a sensory task designed elicit induced oscillatory (beta-band) and evoked responses as well as subtle attentional effects. We reasoned that in addition to measuring these primary responses (which are stable across individuals), neural fingerprinting (i.e., demonstrating that an individual’s OPM-MEG data can be identified from a group, by matching to their SQUID data) would determine whether subtle but repeatable differences between individuals are also measurable across OPM and SQUID-based platforms. Our results showed (1) that primary responses are measurable using both systems with correlations of 0.96 (beta responses) and 0.97 (evoked responses) at the group level; (2) both systems captured significant attentional modulation of the beta rhythm; and (3) fingerprinting was successful in 15 of 15 people using OPM-MEG, 14 of 15 people using SQUID-MEG, and 15 of 15 people when examining cross-platform data. Overall, our results confirm that the two MEG platforms capture similar information on brain function. This is an important step towards proving equivalence between OPMs and SQUIDs, which in turn is required if OPMs are to replace SQUIDs for clinical evaluations.

## 1 Introduction

Magnetoencephalography (MEG) characterises brain function via measurement of magnetic fields generated by neural currents. Mathematical modelling of these fields enables generation of 3D images showing how electrophysiological processes change—moment-to-moment—to support cognition (Baillet, 2017). Conventional MEG systems employ arrays of cryogenically cooled sensors (based on superconducting quantum interference devices—SQUIDs) to measure the (fT-scale) fields from the brain (Hämäläinen et al., 1993). In this way, they provide robust and non-invasive assessment of electrophysiology. However, sensitivity and spatial resolution are limited by the need for a thermally insulating gap between the cryocooled sensors and the head. In addition, the sensor array must be housed rigidly in a “one-size-fits-all” helmet which cannot adapt to head size/shape, and subject movement relative to the (fixed) array degrades data quality. In recent years, a new generation of field sensing technologies—including high-temperature superconductors and optically pumped magnetometers (OPMs)—have become available and ostensibly offer a disruptive change in MEG instrumentation (for reviews seeFaley et al. (2017)andSchofield et al. (2023)). In particular, microfabricated OPMs (e.g.,Sander et al., 2012;Schwindt et al., 2004,^2007^;V. Shah et al., 2007,^2020^;V. K. Shah & Wakai, 2013) allow measurement of the MEG signal closer to the brain (Boto et al., 2017;Iivanainen et al., 2017), enable sensor arrays that adapt to head size (Corvilain et al., 2024;Feys et al., 2022;Hill et al., 2019;Rier et al., 2024), and tolerate movement during scanning (Boto et al., 2018;Holmes et al., 2023). OPM-MEG, therefore, offers a promising alternative to SQUID-MEG.

Despite the promise, OPMs are new technology; their noise levels are higher than SQUIDs and OPM arrays (to date) have fewer measurement channels. There are also several types of OPMs (e.g., with MEG sensitivity along one (Alem et al., 2023), two (Badier et al., 2023;Seedat et al., 2023), or three (Schofield et al., 2024) axes); electronics to control OPMs is still evolving (Schofield et al., 2024) and sensor arrays differ with some systems wearable and others static (like conventional MEG). There is, therefore, a pressing need for techniques and studies that demonstrate equivalence between emergent OPM-based instrumentation and SQUID-based systems. (Indeed, such comparison is critical if OPMs are to replace SQUIDs for clinical evaluation (e.g., in epilepsy).) The aim of such studies is to determine whether different systems capture similar information on brain function; however, methods to demonstrate equivalence are not yet established fully. In almost all OPM-MEG studies to date, comparisons have focussed on measurement of robust signals, including evoked responses (e.g.,An et al., 2022;Borna et al., 2017;Boto et al., 2017;Iivanainen et al., 2019;C. Johnson et al., 2010;C. N. Johnson et al., 2013;Xia et al., 2006, oscillatory processes:Boto et al., 2018;Hill et al., 2020;Iivanainen et al., 2019;Kamada et al., 2015;Rhodes et al., 2023;Sander et al., 2012, epileptogenic features:Feys et al., 2022;Hillebrand et al., 2023, and functional connectivity:Boto et al., 2021;Rier et al., 2023). These “primary responses” tend to be high amplitude, well understood, and repeatable across subjects (or groups). Whilst such studies have been critical in demonstrating the viability of OPM-MEG, more subtle effects that are captured in MEG data—for example, between-subject variance—have been less systematically studied.

Neural fingerprinting is the process by which an individual can be identified from a group, based on their measured brain activity (e.g.,da Silva Castanheira et al., 2021;Sareen et al., 2021). It relies on the fact that, whilst there are features of brain function common across individuals, there is also significant variation between subjects that is meaningful (i.e., not random noise). For example, sensory stimuli lead to a reduction in beta-band (13–30 Hz) neural oscillations during stimulation, followed by an increase (above baseline—termed a “rebound”) on stimulus cessation (Pfurtscheller & Lopes da Silva, 1999). This is measurable in almost all people (and constitutes a “primary response”). However, there are subtle differences between individuals, including the spatial signature of the beta modulation, the relative amplitudes of the beta power reduction and rebound, and the spectral content of the beta signal. If these subtle differences are robust across multiple measures within a single subject, they can form the basis of a neural fingerprint. In addition to task responses, fingerprints can be formed using task-free data, for example, using the spectral content of “resting-state” data from multiple brain locations, or measurements of functional connectivity (Dimitriadis et al., 2023;Haakana et al., 2024). Recent studies have also begun to exploit fingerprinting clinically (Colenbier et al., 2023;Romano et al., 2022;Sorrentino et al., 2021); for example, in Parkinson’s disease (Troisi Lopez et al., 2023) the identifiability of individuals fades as motor symptoms worsen and Parkinson’s disease stages can be decoded (da Silva Castanheira et al., 2024). Between-subject variance in MEG data is, therefore, physiologically meaningful, and fingerprinting is a useful technique to characterise this.

Neural fingerprinting also offers a way to characterise equivalence between MEG systems. Specifically, if an individual’s OPM data can be identified from a group via correlation to their SQUID data (i.e., cross-platform neural fingerprinting), this would support the idea that the two platforms capture similar information relating to between-subject variance. Importantly, this complements what can be learned from primary responses, that is, measurement of primary responses typically tells us about information that is stable across subjects, whereas neural fingerprinting tells us about information that is stable within subjects. Cross-platform metrics could also help to validate fingerprinting itself. For example, it is easy to conceive how identifiability of individuals could be an artefact of system architecture; in conventional MEG, two individuals with identical brain activity may be identifiable, simply because one has a smaller head (and so lower signal strength). By demonstrating identifiability between two systems that use fundamentally different architecture, we would support the neurophysiological origin of MEG fingerprinting. There are a small number of studies suggesting that fingerprinting using OPMs is possible. For example,Rea et al. (2022)used a 90-channel OPM-MEG system to scan two people during a naturalistic handwriting task. Results showed that within-subject correlation was higher than between-subject correlation. Similarly,Rier et al. (2023)measured the test–retest reliability of connectome matrices using a 192-channel OPM-MEG system, demonstrating greater within-subject compared with between-subject correlation. However, neither study directly demonstrated that fingerprinting was possible, nor attempted a cross-platform (SQUID and OPM) measurement.Boto et al. (2021)showed that differences in functional connectivity between individuals were maintained between a 50-channel OPM-MEG and a cryogenic system.Rhodes et al. (2023)measured oscillatory responses to a working memory task and demonstrated that 12 out of 14 individuals could be identified using cross-platform fingerprinting (comparing 174-channel OPM-MEG with 275-channel SQUID-MEG). However, results were confined to the theta band, and the paper did not attempt within-platform fingerprinting. The nascent literature, therefore, suggests that fingerprinting should be possible, both using OPM-MEG alone and between OPM- and SQUID-based instruments.

In this paper, we aim to demonstrate equivalence between SQUID- and OPM-MEG data recorded during a somatosensory attention switching task. This task is known to generate evoked and induced (beta band) primary responses as well as more subtle attentional effects. We will demonstrate that our disparate MEG systems (SQUID and OPM) (1) capture equivalent information on primary evoked/induced responses, (2) enable measurement of the more subtle task-induced attentional modulation, and (3) enable characterisation of between-subject differences, via neural fingerprinting. Specifically, we test a hypothesis that both SQUID and OPM systems will, independently, enable neural fingerprinting, and that the unique neurophysiological signatures of individuals are maintained across the two systems, enabling cross-platform fingerprinting.

## 2 Methods

### 2.1 OPM-MEG system

**Fig. 1.**
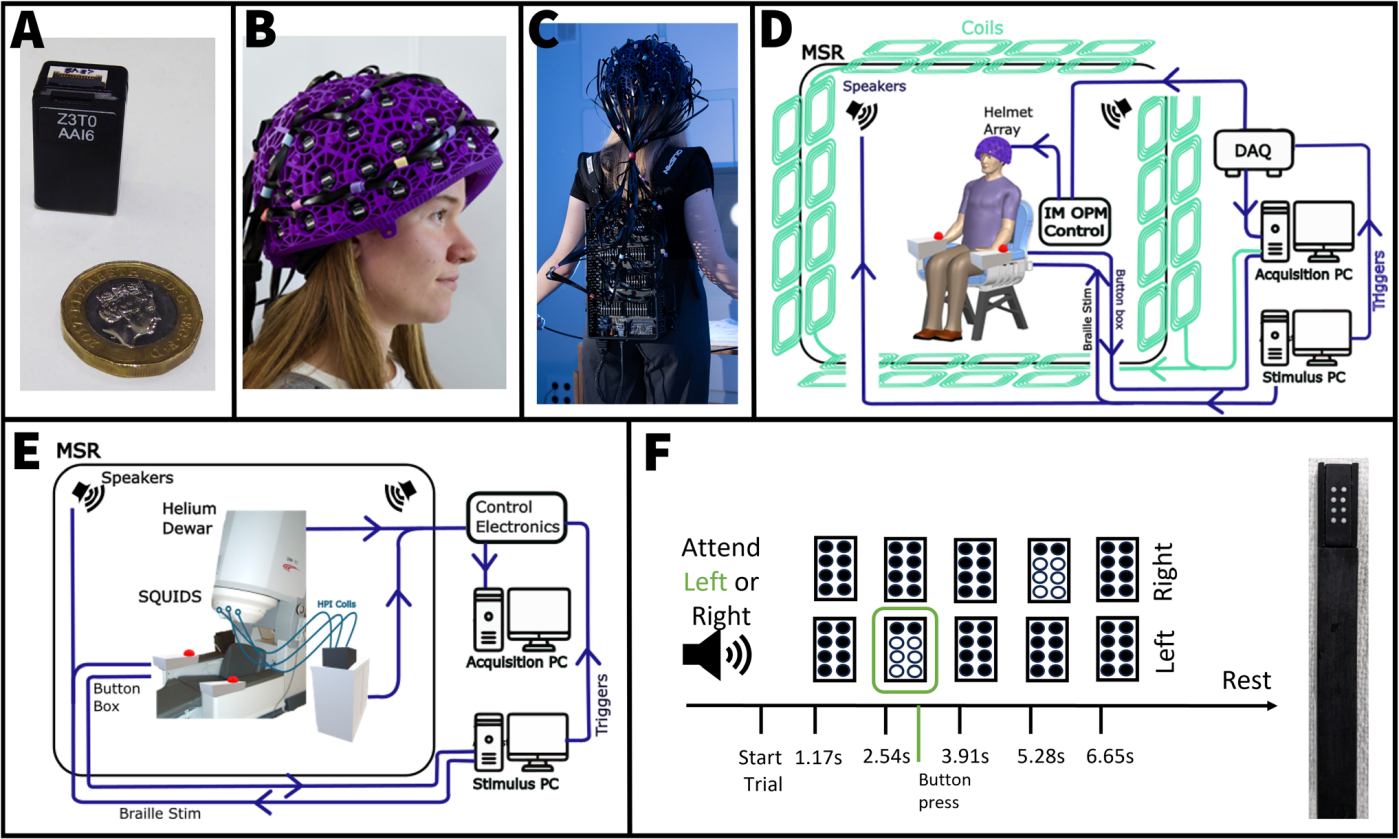
System and paradigm descriptions: (A) A single triaxial OPM sensor. (B) 64 sensors integrated into a 3D printed helmet. (C) The integrated miniaturised electronic control unit. (D) A system schematic for the OPM-MEG system. (E) A system schematic for the SQUID-based instrument. (F) Experimental paradigm and (inset) braille stimulator.

Our OPM-MEG system comprised 64 sensors (3^rd^generation; QuSpin Inc., Colorado, USA), each measuring magnetic fields in three orientations. This allows 3 measurement channels per sensor and so 192 independent field measurements (channels). Each 12.4 x 16.6 x 24.4 mm^3^, 7 g sensor head (Fig. 1A) contained an^87^Rb vapour cell, a laser for optical pumping (tuned to 795 nm—the D1 transition for^87^Rb), on-board electromagnetic coils for field control within the cell and two photodetectors for signal readout. To enable triaxial measurement, the laser was split into two orthogonal beams by a beam splitter (Boto et al., 2022;V. Shah et al., 2020) and projected through the cell in the*x*(beam 1) and*y*(beam 2) orientations. Field measurements within an OPM are most sensitive when made perpendicular to the direction of the laser. Consequently, field measurements in the*y*and*z*orientations were made using beam 1, and measurements in the*x*and*z*orientations were made using beam 2. Combining the four metrics provides complete assessment of the magnetic field vector through the cell.

All sensor heads were mounted in a 3D printed helmet (Fig. 1B) (Cerca Magnetics Limited, Nottingham, UK) which distributed sensors evenly to provide (approximate) coverage of the entire cortex. The helmets were designed in multiple sizes (to accommodate different head sizes), and so that each sensor head could be located into a “slot.” Once sited, the relative locations and orientations of the sensors could be determined using the computer-aided design (CAD) file for the helmet. The helmet was also designed with an open lattice surrounding the sensors to enable efficient convection of heat generated by the sensors.

OPMs require the ability to tightly regulate the cell/vapour and laser temperatures, locate and lock the laser output to the^87^Rb D1 resonance, control onboard coils (to null magnetic fields within the cell, and produce the required “modulation” field to enable directional sensitivity;Cohen-Tannoudji et al., 1970) and read photodetector responses via lock-in detection. This control was enabled via an integrated miniaturised (IM) electronics unit (Schofield et al., 2024;*“NEURO-1”*, QuSpin, Colorado, USA). The unit was designed to be small, compact, and wearable as a backpack (0.36 x 0.2 x 0.06 m^3^and weighing 1.8 kg) (Fig. 1C). Each sensor was connected to the electronics unit by a 90 cm ribbon cable and controlled by a single electronics card. These cards were grouped into modules and the digital output of each module was sent to a multiplexer. A network card passes the output from all sensors, via ethernet, to a decoder (DAQ—sbRIO9637, National Instruments) which then passes the signal to an acquisition PC where data are recorded at a 375 Hz sampling frequency. Sensors were operated in “closed-loop” (in which on-board-sensor electromagnetic coils use negative feedback to compensate for any changes in local field to increase the dynamic range of the sensors).

The system components (helmet, OPM array, and IM electronics) were housed inside a magnetically shielded room (MSR). The decoder and acquisition computer were kept outside the MSR to reduce magnetic interference. The MSR (MuRoom, Magnetic Shields Limited, Kent, UK) comprised four layers of material with a high magnetic permeability (MuMetal) to reduce the DC and low-frequency environmental magnetic field, and a single layer of material with high electrical conductivity (copper) to reduce high-frequency environmental fields. The MuMetal layers were equipped with degaussing coils to reduce any DC field inside the room caused by magnetisation of the walls (Altarev et al., 2015). A “Matrix Coil” system (Holmes et al., 2023), comprising 94 independently controllable square (88 x 45 cm x 45 cm and 6 x 35 cm x 35 cm panels) electromagnetic coils (arranged mainly as 4 x 4 grids of coils integrated into all walls, ceiling, and floor of the MSR), was also available to generate known spatial and temporal variation of magnetic field inside the room. The matrix coil was controlled by the acquisition PC and reduced the DC background field over the OPM-MEG helmet to <1 nT. A separate computer controlled the experimental stimuli delivered to the participants. A schematic of this system is given inFigure 1D.

### 2.2 SQUID-MEG system

For SQUID-MEG we used a 275-channel cryogenic system (CTF, Vancouver, BC, Canda), housed inside an MSR with 2 layers of mu-metal and 1 layer of aluminium (Ak3b, Vacuumschmelze, Hanau, Germany). Each SQUID-MEG sensor comprised a 5 cm baseline axial gradiometer flux transformer, magnetically coupled to a (remotely located) SQUID. The system, therefore, measures the gradient of the magnetic field oriented approximately radial to the scalp surface. The system also contains a reference array with 29 SQUID-based sensors located distal to the head. This reference array enables measurement of ambient magnetic interference in the room. Signals from the scalp and reference array were combined using a synthetic third-order gradiometer approach to reduce the effects of magnetic interference (Vrba & Robinson, 2001). The SQUIDs are controlled by an electronics unit located outside the MSR. All data were collected using an acquisition PC, at a sampling frequency of 600 Hz. A separate computer controlled the experimental stimuli. A schematic of this system is given inFigure 1E.

### 2.3 Paradigm and subject recruitment

All experiments were approved by the University of Nottingham Faculty of Health Science Research Ethics Committee (approval number H16122016). In total, 16 participants (all of whom had experience of being participants in MEG studies) each took part in 4 MEG scans and 1 anatomical MRI scan, the latter being used for source modelling. Of the 16 participants, 8 identified as male, 8 as female, their mean age was 27 ± 5 years and 15 self-identified as right handed. The MEG scans were split into four sessions, two in the morning and two in the afternoon, on the same day. Of the 16 participants, 8 followed “schedule A” (OPM-MEG in the morning, and SQUID-MEG in the afternoon) and the other 8 followed “schedule B” (SQUID-MEG in the morning and OPM-MEG in the afternoon). MEG data were collected using the systems described above. MRI data were acquired using a 3 T Philips Ingenia scanner running an MPRAGE sequence (1 mm^3^resolution). In total, the study acquired 64 MEG and 16 MRI datasets.

The experimental paradigm was adapted from a previous study (Bauer et al., 2006) and involved sensory stimulation of the left and right index fingers. Specifically, we used 2 independently controllable “braille” stimulators (Metec, Germany), each comprising 8 “pins” arranged in a 4 x 2 matrix (seeFig. 1F(inset)). Each pin could be driven independently by a piezoelectric crystal, forcing it “up” (stimulating) or “down” (not stimulating). By manipulating which pins were up and down, several different “braille-like” tactile patterns could be applied to the participants fingers. Here we used two patterns: a*non-target*in which all eight pins were up, and a*target*in which only the top two pins were up.

A single experimental trial began (at time*t=0 s*) at the end of an auditory attentional cue telling participants to either “attend left” or “attend right.” Following this, at*t=1.17 s*a series of 5 braille patterns were presented, at intervals of 1.37 s, to both index fingers. The stimuli lasted 370 ms with a 1 s pause between patterns. Participants were asked to respond, with a button press using both their left and right thumbs, if the target pattern (of only two raised pins) was presented to the attended hand (this is shown diagrammatically inFig. 1F). The button response was recorded using an MEG-compatible response box (Current designs Inc., Philadelphia, PA). Each trial ended with a 7-s rest period. To delineate stimulus timing, signals (termed “triggers”) were sent from the stimulation computer (which was driving the braille stimulators) to the data acquisition unit. Separate triggers identified the attentional cue, braille stimulation (targets and non-targets), and button response. A single experiment consisted of 80 trials. In total there were 80 target patterns and 320 non-target patterns presented to both the attended and non-attended hands (i.e., the probability of a target was 0.2). Some trials could contain multiple targets, whilst others may contain none. The total length of each experiment was 22 min, 30 s. This paradigm was known to generate multiple neuromagnetic effects including somatosensory evoked responses to each stimulus, a beta (13–30 Hz) band-induced modulation, and a more subtle attentional effect whereby beta oscillatory amplitude was expected to reduce in the primary sensory area contralateral to the attended hand (Bauer et al., 2006).

### 2.4 Coregistration of MEG data to brain anatomy

To allow for MEG data modelling, we require knowledge of the location and orientation of the sensors relative to the brain anatomy—termed coregistration. For this we used two independent techniques, which have each become standard practice for OPM- and SQUID-MEG recordings:

- *OPM-MEG coregistration*: immediately following data acquisition, a structured light camera (Einscan H, SHINING 3D, Hangzhou, China) was used to gather a 3D digitisation of the participants face, head, and helmet. We acquired two structured light scans, one with the helmet in place (termed the face/helmet digitisation) and a second with the helmet removed and a swimming cap worn by the subjects to flatten their hair (termed the no-helmet digitisation). A third digitisation of the head/face was extracted from the MRI. Initially, a helmet CAD file (which included relative sensor locations and orientations) was fitted to the face/helmet digitation using point-to-point fitting. The helmet/face digitisation was then fitted to the non-helmet digitation (again using point-to-point fitting). Following this, the no-helmet digitisation was fitted (using MNE-python;Gramfort et al., 2013) to the MRI-derived scalp surface (extracted using FreeSurfer;Fischl, 2012). By combining these coordinate transformations, it was possible to co-register the sensor locations and orientations to brain anatomy in the MRI (Hill et al., 2020;Zetter et al., 2019).
- *SQUID-MEG coregistration*: Prior to SQUID-MEG data acquisition, three head position indicator (HPI) coils were attached to the head at the nasion and pre-auricular locations. These coils were energised throughout the scan and a magnetic dipole model of the measured field used to determine their location within the MEG helmet. This enabled real-time head movement tracking and knowledge of the HPI coil locations relative to the MEG sensor array. Immediately following the recording, the 3D locations of the HPI coils (with respect to the head and face shape) were derived using a 3D digitiser (Polhemus, Vermont, USA). The digitised head shape was fitted to the equivalent surface extracted from the MRI scan (again by using FreeSurfer and MNE-python;Fischl, 2012;Gramfort et al., 2013). Combining this with knowledge of the sensor locations relative to the HPI coils provided a complete coregistration of SQUID sensor geometry to brain anatomy. Note that this approach to coregistration was chosen as it reflects the “standard” practice for SQUID-MEG recording.

### 2.5 Data preprocessing

All data were organised according to the Brain Imaging Data Structure (BIDS) for MEG (Niso et al., 2018). For OPM-MEG, a power spectral density plot was created (using Welch’s method;Welch, 1967) for all channels, and any channels with an average noise floor lower than 5 fT/√Hz, in the 60–80 Hz band, were removed. (The 60–80 Hz band was chosen to generate an estimate of the noise inherent to a sensor. Note this is distinct from magnetic interference which tends to manifest at low frequency, and also distinct from the powerline (50 Hz) and its harmonics.) This ensured removal of channels that were recording little or no signal (i.e., not operational). Following this, all PSD plots were subject to visual inspection, and any channels showing high noise (above ~20 fT/√Hz in the 60–80 Hz band) were also removed. Homogeneous field correction (HFC) (Tierney et al., 2021) was applied to remove interference that manifests as a spatially uniform field across the OPM array. Following this, data were filtered to two frequency bands: broad band (1–100 Hz) and the beta band (13–30 Hz). Both datasets were segmented into trials and any bad trials (where one or more channels had a maximum peak-to-peak amplitude higher than 4 pT) were removed. For SQUID data, interference was reduced via synthetic third-order gradiometry (Vrba & Robinson, 2001). All channel data and trials were inspected visually by an experienced MEG operator and any channel or trial with high noise was removed. (Note: all frequency filters used default settings in MNE-python and were based on a FIR filter, the order of which was given by the filter length (6.6 times the reciprocal of the shortest transition band) minus 1.)

### 2.6 Images of brain function

All data processing was carried out using MNE-python (Gramfort et al., 2013). For both the SQUID-MEG and the OPM-MEG datasets, we derived two separate functional images.

- **Beta modulation:**Data were filtered into the beta band and segmented into 1.2 s windows (i.e., 0 s ≤ t ≤ 1.2 s) relative to tactile stimulus onset. We then used a linearly constrained minimum variance (LCMV) beamformer for source localisation. Covariance matrices were calculated for “early” (0.2 s ≤ t ≤ 0.5 s) and “late” (0.75 s ≤ t ≤ 1.05 s) time windows and beamformer weights derived using data covariance averaged across both. (This covariance matrix was regularised with a regularisation parameter equal to 5% of the maximum singular value of the unregularised matrix.) Source orientation for each voxel was chosen to be the orientation of maximum projected source power. Having derived the beamformer weights for each voxel, we contrasted the early and late time windows to generate a pseudo-T-statistical image showing the spatial distribution of beta modulation across the brain.
- **Evoked responses:**Data were filtered into the 5–40 Hz band and again segmented into 1.2 s windows relative to tactile stimulus onset. We then used a minimum norm estimate (MNE) for source localisation. (Note, we chose not to use a beamformer for evoked responses since the paradigm involved bilateral and simultaneous stimulation of the left and right index fingers, which likely drives correlated evoked responses in left and right sensorimotor areas, which beamforming will supress.) Data were averaged over trials and, for each voxel, the minimum norm estimate of source power generated (along all three orthogonal orientations; these were then summed in quadrature to generate a single time course per voxel). Images of evoked power were generated for the peak in the evoked response time course.

For both images a volumetric approach was taken. The brain volume was divided into 5 mm cubic voxels. A forward solution was calculated for all voxels using a dipole approximation and a single shell head model (Nolte, 2003). These images were derived for all four datasets in all subjects; they were co-registered to the Montreal Neurological Institute (MNI) (Collins et al., 1998) standard brain using MNE-python.

### 2.7 Time–frequency dynamics

For the locations of peak task-induced beta modulation in left and right primary sensory cortex, we derived a time–frequency representation of the MEG signal. Here, beamformer weights were derived using covariance calculated using all available broadband data (regularised as above). The weights were then used to derive a broadband estimate of electrophysiological activity at the locations of interest (a virtual electrode). These data were frequency filtered into overlapping bands (each 1 Hz bandwidth) between 1 and 100 Hz and, for each band, a Hilbert transform used to derive the analytic signal. The absolute value of the analytic signal was then calculated to generate the instantaneous amplitude of the band-limited signals—termed the Hilbert envelope. These were then averaged over trials to give a single trial averaged time course per frequency band,A(t,f). For all frequency bands, we calculated a baseline oscillatory amplitude,B(f)in the 12 s ≤ t ≤ 13.5 s time window (relative to trial onset). We then computed the final TFS asR(t,f)=((A(t,f)−B(f))​/​B(f), which represents the relative change in oscillatory amplitude for all frequencies. In addition to the TFS, we derived the envelope of beta oscillations throughout the task trial. Here, beamformer weights were computed using beta-band data. A beta band virtual electrode was calculated, and its Hilbert envelope derived. This was then averaged across trials for each subject.

For the locations of maximum evoked power in left and right primary sensory cortex, we also derived a time course of the evoked power. Specifically, from the source space MNE reconstruction, we took the data averaged over all stimulus presentations and found the peak voxel from the evoked response (note the timing of this differed slightly across subjects, as we chose the peak time point for each subject). We took the time courses of evoked response in all three orthogonal orientations for this single voxel and added them in quadrature to yield a single time course of estimated evoked power in the 0 to 1.2 s time window following somatosensory stimulus presentation.

### 2.8 Contrasting primary responses

Initially we aimed to quantify the similarity between primary responses measured using the two MEG systems (SQUID and OPM). To this end, we took the beta envelope time courses and averaged them over subjects (two runs per subject) for each MEG system, to generate group average time courses. We then computed the correlation between these averaged envelopes to quantify the similarity between MEG systems. We then reasoned that if the OPMs and SQUIDs were measuring different information (at the group level), this value would differ from an empirical null distribution. To derive the null, we took all the time courses (15 OPM and 15 SQUID) and randomised their order, resulting in two new groups in which SQUID and OPM time courses were mixed. Time courses were averaged across these new “sham” groups and the correlation re-derived. This procedure was repeated 10,000 times to form the null distribution. We then quantified where the true value of between-system correlation lies in relation to the null, assuming that it would fall in the tails of the null distribution if there was a significant difference between systems. This analysis was repeated for the evoked response time courses.

### 2.9 Contrasting attentional effects

Previous studies (Bauer et al., 2006;Rivero et al., 2024) have shown that, in addition to the primary responses, our paradigm is likely to elicit modulation of the beta envelope in sensory cortex, around the time of the attentional cue. Specifically, when subjects are told to attend to their right hand, the beta amplitude drops in left primary sensory cortex (compared with when attending their left hand). Similarly, attending the left hand would generate a relative drop in beta amplitude in right primary somatosensory cortex. To measure this, we first plotted the beta envelopes in left and right primary sensory cortex for two conditions: attend left and attend right. For both regions, we measured the difference in mean beta amplitude between conditions (attend left—attend right) in a time window -0.8 ≤ t ≤ 1 relative to the end of the attentional cue. We expected these beta differences to be positive in left sensory cortex (where attending*right*causes a drop in beta) and negative in right sensory cortex (where attending*left*causes a drop in beta). To assess statistical significance, we contrasted the beta differences in the two regions using a Wilcoxon sign-rank test. This analysis was equivalent for both OPM and SQUID systems.

### 2.10 Neural fingerprinting

To implement neural fingerprinting, we considered only the beta band effects and took into account both the pseudo-T-statistical images and the TFSs from the peak location in primary sensory cortex. Our study had a total of 64 datasets and for each pair of datasets we calculated:

(1) The correlation between the two pseudo-T-statistical images in MNI space
(2) The correlation between the TFSs derived in the primary sensory cortex.

For all the measures that follow, these two metrics were averaged together (but see alsoSupplementary Information).

The principle of neural fingerprinting is that within-subject correlation should be higher than between-subject correlation. This means that if we take a group of participants and scan each of them twice, and then correlate data from experiment-1 in subject 1, with data from experiment 2 in all subjects, the highest correlation value should be generated by the subject 1-to-subject 1 comparison. In this way, individuals become identifiable. Here, however, this is complicated by the fact that we have not two, but four experiments per subject. This permits multiple comparisons, within/between subjects, and within/between platforms. The possible comparisons between datasets are shown graphically inFigure 2, for the simplified case of two subjects.

**Fig. 2.**
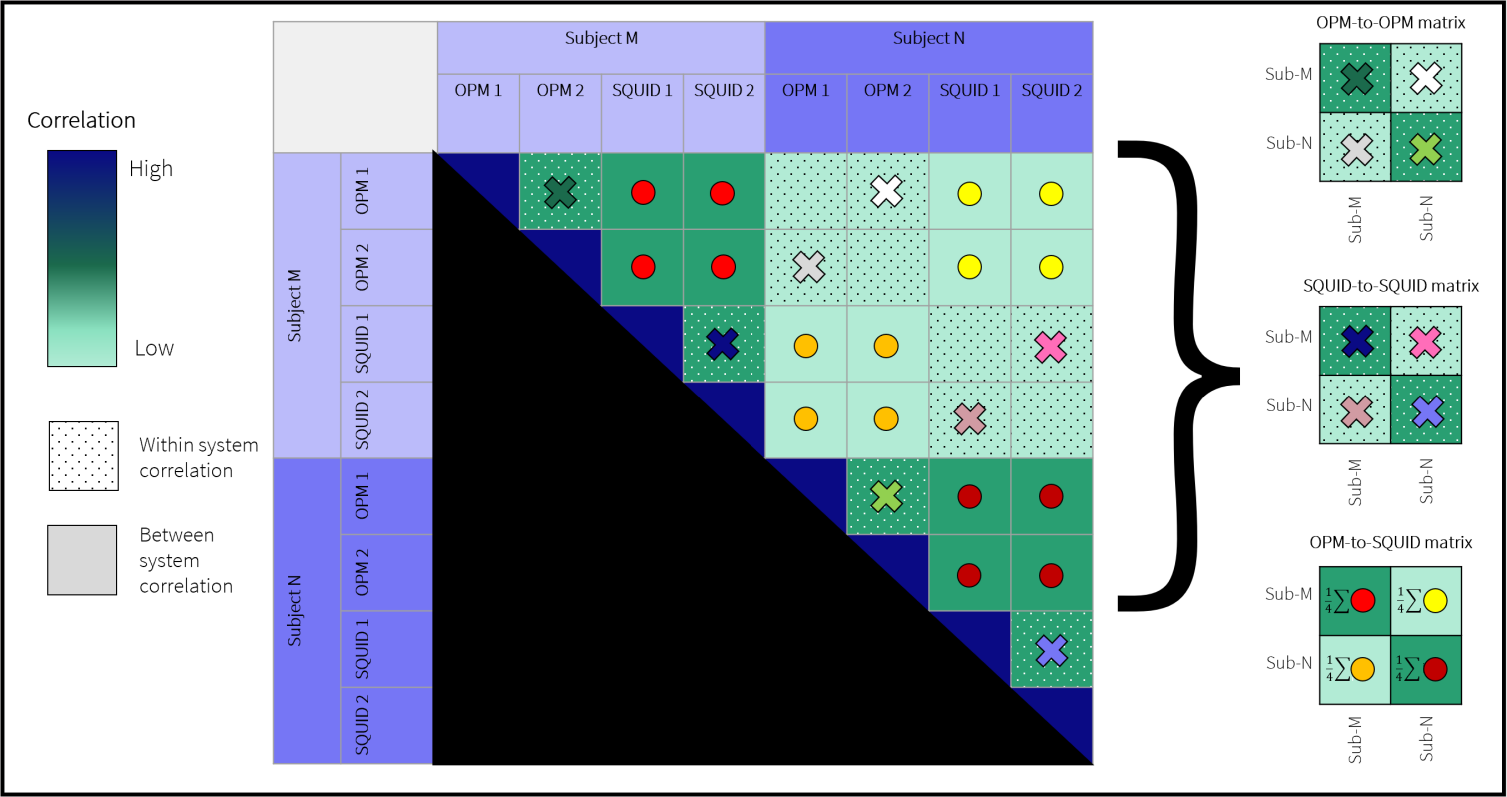
Data analysis for neural fingerprinting: the matrix shows, for two subjects, the possible correlation values that can be derived between the four acquired datasets, and how these values are used to construct the three fingerprinting matrices.

**Within platform:**For fingerprinting using the OPM system, there is only a single within-subject OPM-to-OPM comparison for each subject (shown by the dark green cross for subject M, and the light green cross for subject N); these both compare experiment 1 with experiment 2. (Note: comparing experiment 1 with experiment 1 would be meaningless within a subject, since the correlation would be one.) There are four possible between-subject OPM-to-OPM comparisons, but only two of these can be considered independent. We, therefore, chose to only compare experiment 1 in subject M with experiment 2 in subject N (white cross), and experiment 2 in subject M with experiment 1 in subject N (grey cross). This ensured equivalence for the within- and between-subject measures. Similarly, when fingerprinting using the SQUID data, we derived within- and between-subject comparisons by only comparing experiment 1 with experiment 2. For SQUIDs, the dark and light blue crosses inFigure 2represent within-subject comparisons and dark/light pink crosses represent between-subject comparisons.

**Cross-platform:**When considering cross-platform fingerprinting, there are four possible within-subject comparisons for subject M (red dots); four within-subject comparisons in subject N (dark red dots); four between-subject comparisons using OPM data in subject M and SQUID data in subject N (yellow dots); and finally four between-subject comparisons using SQUID data in subject M and OPM data in subject N (orange dots). Since in all cases the number of comparisons was the same, we averaged all four values together to construct the final fingerprinting matrices. This again ensured equivalence for all the derived matrix values.

The result of this was three fingerprinting matrices, representing*within OPM*,*within SQUID*, and*OPM-to-SQUID*comparisons (these are shown schematically on the right-hand side ofFig. 2). For each matrix (with dimensions N_subjects_x N_subjects_), the on-diagonal elements represent within-subject comparison, and off-diagonal elements represent between-subject comparison. For successful fingerprinting, we expect the on-diagonal elements to be larger than the off-diagonal elements.

Having derived these matrices, we conducted two analyses. First, for each matrix row, we found the value of the largest average correlation to see if a subject could be successfully identified from the group (i.e., does the largest value occur along the leading diagonal). Second, we averaged the on- and off-diagonal elements of the matrix and measured the difference between these mean values. We then reasoned that if neural fingerprinting was not possible, the order in which the subjects’ data were presented would be meaningless. We performed a permutation test by randomising the subject labels, rederiving the matrix, and again measuring the difference between the averages of the on- and off-diagonal elements. This randomisation step was repeated 100,000 times to create an empirical null distribution. This enabled calculation of an empirical p-value representing the probability that the real difference between within- and between-subject correlation could occur by chance.

## 3 Results

One subject was removed from the cohort due to a problem with triggers in the SQUID-MEG. This left a total of 15 participants in our final analysis (7 male, 8 female, mean age 27 ± 5 years and 14 right handed). We removed 5 ± 2 and 8 ± 6 channels from the SQUID and OPM data, respectively, due to high noise or channel failure. We removed 2 ± 2 and 5 ± 5 trials, also due to high noise or artefacts. (These values represent mean ± standard deviation across subjects.)

In general participants performed the task well, with 96 ± 8% of target stimuli correctly identified during the OPM-MEG experiments and 98 ± 3% during SQUID experiments (in both cases, numbers represent mean ± standard deviation across subjects). We had deliberately made the task easy compared with similar experiments in previous papers (Bauer et al., 2006;Rivero et al., 2024) to minimise learning effects across the four runs of the same experiment. This is reflected in the high success rates.

### 3.1 Primary responses

**Fig. 3.**
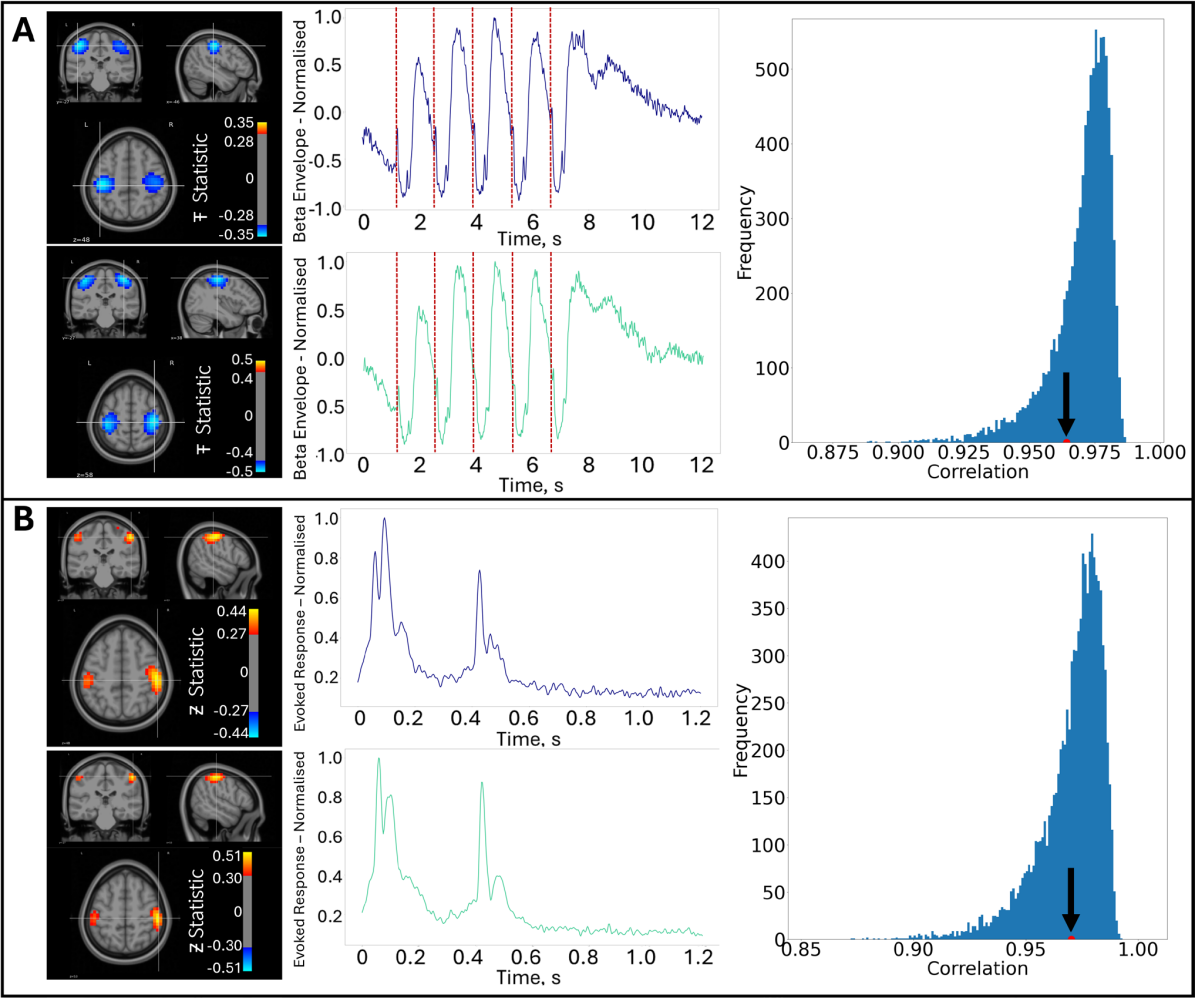
Primary responses to braille stimulation: (A) Images show the spatial signature of beta modulation (left panel, blue overlay) plotted on the MNI brain. Time courses show beta modulation throughout the task trial with the sensory stimuli marked by the red dashed lines. In both cases, the upper panel shows OPM data and the lower panel shows SQUID data. The right-hand panel shows the correlation between SQUIDs and OPMs (black arrow) plotted alongside a null distribution. (B) Similar to (A) but showing evoked response data—note we show the evoked response to a single sensory stimulus, with peaks representing the onset and offset of stimulation.

Figure 3Ashows the group averaged pseudo-T-statistical images (left panel) and time courses (centre panel) for beta modulation. Data are averaged across both experiments and subjects; the upper panel shows OPM data and the lower panel shows SQUID data. In the time courses, stimulus onsets are denoted by the red dashed lines. As expected, for both systems, the beta modulation is localised to the primary sensory cortices, and each sensory stimulus generates a transient drop in beta amplitude followed by a short rebound. The correlation in the group averaged beta time courses between the two systems was 0.96. The right-hand panel shows that this correlation (marked by the arrow) was not different from the empirical null distribution.Figure 3Bshows similar data for the evoked responses. Clear evoked responses are observable for sensory stimuli with a correlation between systems (at the group level) of 0.97. Again we see no measurable difference between this correlation and the null distribution. Both results, therefore, imply that, at the group level, OPMs and SQUIDs capture similar primary responses.

### 3.2 Attentional shifts

**Fig. 4.**
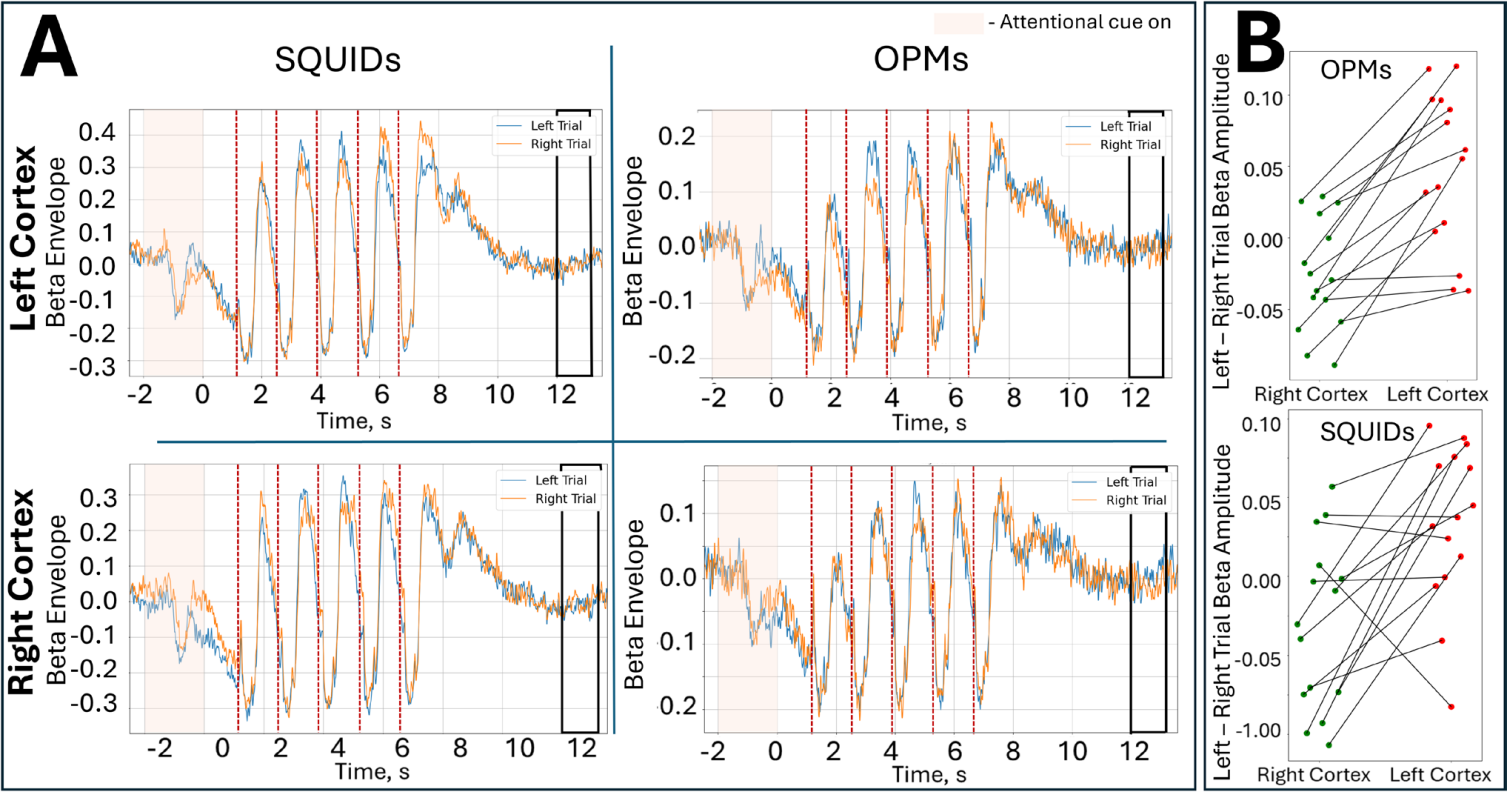
Attentional modulation of beta amplitude: (A) Beta-band time courses for the left (top) and right (bottom) primary sensory cortices. Data from the OPM and SQUID systems are shown in the left and right columns, respectively. Red dashed lines show the onsets of the tactile stimuli and the shaded window marks the time period where the participant is told which hand they should attend to. In all cases, the orange line represents a trial in which attention was directed right, and the blue shows left-attended trials. (B) Each data point represents the difference in beta envelope between left- and right-attended trials, in the -0.8 < t < 1s time window following the attentional cue for a single subject. Data for OPMs are shown in the upper panel and SQUIDs are shown in the lower panel. In both cases, left and right sensory cortices are shown in the right and left halves of the graph. Note that these differences tend to be negative in the right hemisphere and positive in the left hemisphere, for SQUIDs and OPMs.

Figure 4Aagain shows beta band time courses, from the left and right primary sensory regions, for OPMs and SQUIDs. Here, the orange trace shows trials where attention was directed to the right hand and the blue trace shows trials where attention was directed to the left hand (data averaged across subjects). In left sensorimotor cortex, around the time of the sensory cue, the literature (Bauer et al., 2006;Rivero et al., 2024) suggests that we should expect a drop in beta amplitude when subjects attend right, relative to when they attend left (i.e., the orange line should dip beneath the blue line). Similarly, in right sensorimotor cortex, we would expect a drop in beta amplitude when subjects attend left (i.e., the blue line should dip beneath the orange line). This effect was observable in both OPMs and SQUIDs, providing additional evidence of similarities between systems. This is formalised inFigure 4B. Here, each data point represents the difference in beta amplitude between attend left and attend right conditions, in a time window immediately following the attentional cue (black lines link data points for all individual subjects). For both SQUIDs and OPMs, there were significant (*p*= 0.00202 and*p*= 0.000014 respectively; Wilcoxon sign rank test) differences between the left and right primary sensory cortices with beta amplitude differences being negative (on average) in the right hemisphere and positive (on average) in the left hemisphere.

### 3.3 Neural fingerprinting

**Fig. 5.**
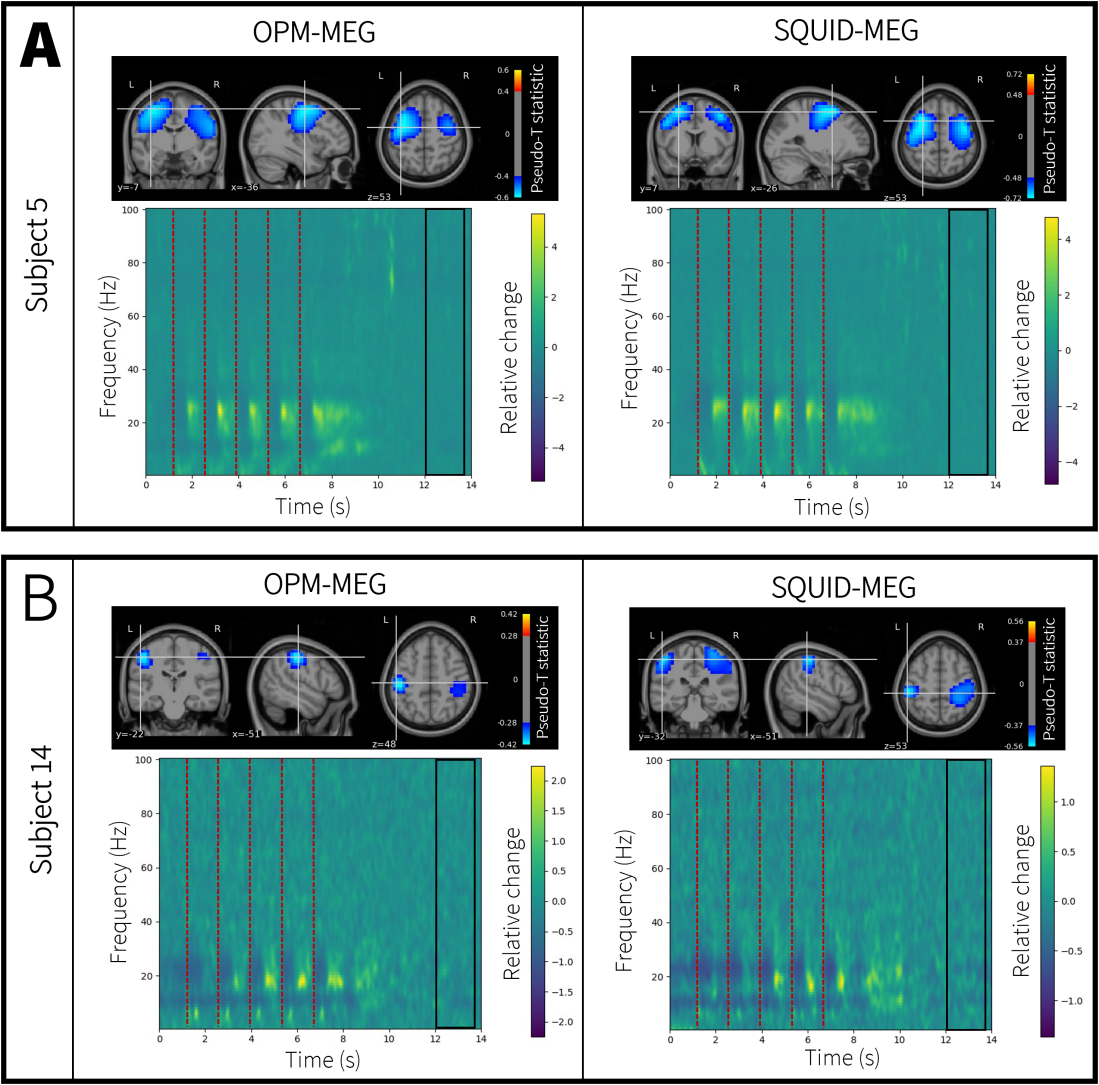
The basis of fingerprinting: Data from two representative subjects. The left panel shows OPM-MEG; the right panel shows SQUID-MEG. (A) Shows subject 5 and (B) shows subject 14. In all cases, the images show an MRI scan with the spatial signature of beta modulation (i.e., the pseudo-T-statistical image) overlaid in blue. The TFS from the left sensory cortex is also shown. Here blue represents a decrease in oscillatory amplitude relative to baseline. Yellow reflects an increase. Notice that whilst features are largely similar, there are differences between the two subjects that are maintained across the two scanner types.

Figure 5shows representative data from two individual participants. In both cases, pseudo-T-statistical images of beta modulation and a TFS from left primary sensory cortex are shown. The left panel shows OPM-MEG data, and the right panel shows SQUID-MEG data. In both subjects, the largest beta modulation (represented by the blue overlay) localises to the primary somatosensory cortex. In the TFS, blue represents a decrease in oscillatory amplitude relative to baseline, and yellow represents an increase. (The baseline window is indicated by the black box.) In agreement with the group average data (above), each of the five tactile stimuli (marked in theFig. 5with the dotted lines) elicits a transient drop in beta amplitude followed by a brief rebound (above baseline) following stimulus cessation. There are also low frequency increases in amplitude immediately following each tactile stimulus, which likely reflect the evoked response. Data appear high quality in both systems.

Although there is a marked similarity between the two subjects, there are also observable differences. For example, in the TFS for subject 5, the rebounds following each of the five tactile stimuli are approximately equal in amplitude. However, in subject 14, rebound is consistently larger for later stimuli compared with the earlier stimuli. Similarly, the spatial signature of the beta modulation appears to be more widespread in subject 5 than in subject 14. It is these between-subject differences, which are shown to be consistent across two independent MEG scanner platforms, that form the basis of neural fingerprinting.

**Fig. 6.**
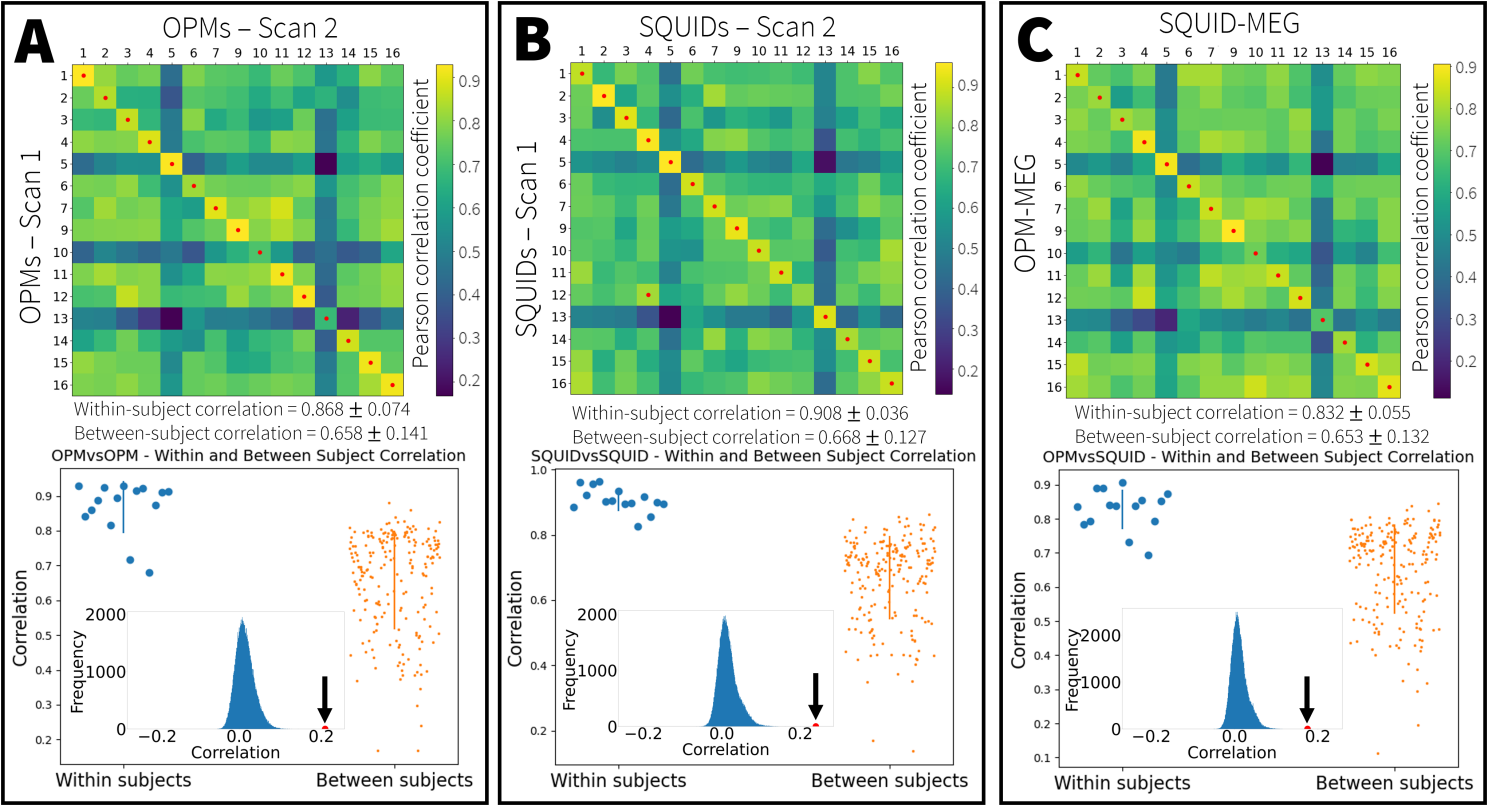
Neural fingerprinting: (A) OPM-based fingerprinting. (B) SQUID-based fingerprinting. (C) Cross-platform fingerprinting. In all cases the upper panel shows the fingerprinting matrix where element[i,j]represents correlation between subjectiand subjectj. The on-diagonal elements represent the within-subject correlation and off-diagonal elements represent between-subject correlation. The red dots show the largest correlation value for each row in the matrix; in cases where these dots fall on the leading diagonal, fingerprinting is successful in the corresponding subject. In the lower panel, the blue points show the correlation values for the on-diagonal elements and the orange points show the correlation values for the off-diagonal elements. The mean and standard deviation are also shown. The inset graph shows the difference in mean on- and off-diagonal elements for the real data (marked by the arrow), in relation to an empirical null distribution.

Figure 6A(upper panel) shows the fingerprinting matrix for the OPM data only. Recall each matrix element represents averaged correlation between the pseudo-T-statistical image and the TFS. Matrix element[i,j]represents the correlation between subject*i*(experiment 1) and subjectj(experiment 2). Thus, diagonal elements show within-subject correlations and off-diagonal elements show between-subject correlation. For all 15 subjects (each row in the matrix), the highest value of correlation was found for within-subject comparison (i.e., for each row, the highest value (denoted by the red cross) is found within the corresponding column). This means that all 15 subjects were correctly identified using the fingerprinting method. In the lower panel, the blue data points show the values of the diagonal elements of the fingerprinting matrix, and the orange points show the off-diagonal elements. The mean and standard deviations for both are also shown. The inset graph shows the difference between the averages of the diagonal and off-diagonal elements (marked by the arrow) compared with an empirical null distribution, demonstrating that within-subject correlation is significantly higher than between-subject correlation. (Note that these metrics do not demonstrate subject identifiability*per se*, rather they simply show that there are features in MEG data which are unique to specific subjects (and not shared between subjects)).Figure 6Bshows the same information for the SQUID data. Here 14 of the 15 subjects were correctly identified using fingerprinting, and the difference between within- and between-subject correlation remains significant.

Figure 6Cshows the cross-platform fingerprinting matrix. Whilst the matrix has the same structure to those inFigure 6A and B, recall that for cross-platform fingerprinting, matrix element[i,j]represents the average of four possible comparisons (seeFig. 2). Here again, all 15 subjects were successfully identified and the difference between the on- and off-diagonal elements of the matrix is again significant. This is a key result which demonstrates that the OPM system is generating similar information on inter-subject variability as the SQUID system, across this subject cohort. This will be discussed further below.

It is also noteworthy that across the three fingerprinting matrices there is significant similarity; indeed Pearson correlation values between the three matrices were computed as$r_{OPM-SQUID}=0.78$($p=6^{-47}$),$r_{OPM-X-platform}=$0.89($p=5^{-77}$), and$r_{X-platform-SQUID}=0.85$($p=7^{-65}$).

## 4 Discussion

The aim of this study was to demonstrate that SQUID- and OPM-based MEG platforms generate similar information on brain function. There have been several studies demonstrating the efficacy of OPMs for MEG (examples include:An et al., 2022;Borna et al., 2017;Boto et al., 2017,^2018^,^2021^;Feys et al., 2022;Hill et al., 2020;Hillebrand et al., 2023;Iivanainen et al., 2019,^2020^;C. Johnson et al., 2010;C. N. Johnson et al., 2013;Kamada et al., 2015;Rhodes et al., 2023;Rier et al., 2023;Sander et al., 2012;Xia et al., 2006and for reviews see also:Brookes et al., 2022;Schofield et al., 2023). However, most of these studies have probed the ability to use OPMs to measure primary responses to stimuli. Here we contrasted a 192-channel OPM-MEG scanner and a 275-channel SQUID-based instrument, complementing traditional metrics of primary responses with neural fingerprinting. We showed that the beta and evoked responses to somatosensory stimulation are highly correlated across OPM and SQUID-based systems; indeed a statistical analysis showed that the group level data from the two platforms were indistinguishable, when compared with an empirical null distribution. We further showed that both systems were sensitive to subtle more subtle attentional effects induced by our paradigm. Using neural fingerprinting, we reasoned that if we could identify the OPM-MEG data recorded from a specific individual, using only their SQUID-MEG data as a reference, then it is likely that the two independent MEG systems are capturing similar information about features that differentiate individual brain function. Our results showed that this is possible. Within-subject correlation of measured brain function, measured across the 2 platforms, was significantly higher than between-subject correlation, and 15 out of 15 participants had their OPM data correctly identified, using SQUID data as a reference. Combined, these results suggest cross-platform equivalence and show that OPMs and SQUIDs not only both measure primary stimulus responses, but also capture similar between-subject variance.

We chose to limit our fingerprinting analysis to just the oscillatory effects. However, in ourSupplementary Information, we also show fingerprinting results for the evoked responses. Here, we conducted cross-platform and within-platform fingerprinting (using an equivalent method to that used for the oscillatory responses (e.g.,Fig. 2) and taking into account both the evoked response time courses averaged across sensory stimuli (i.e., similar to the time courses shown inFig. 3) and the functional images showing the spatial signature of the evoked responses). Interestingly, evoked response fingerprinting was less successful than that based on beta oscillations; our cross-platform metric correctly identified 10 of 15 subjects; within-platform SQUID-based fingerprinting identified 9 of 15 subjects, whilst within-platform OPM-based fingerprinting identified just 5 of 15 subjects. This is in contrast to fingerprinting based on beta oscillations, where the equivalent 3 metrics were 15/15, 14/15, and 15/15. Whilst the reason for this remains unknown, it is tempting to speculate that the oscillatory response, which comprises top-down influence of the broader sensorimotor network on primary sensory cortices and incorporates attentional effects, exhibits a greater between-subject variance than evoked response, which reflects a simpler physiological process. Here we concentrate on fingerprinting based on functional images, time–frequency spectra, and evoked responses. However, there are many other metrics including connectome matrices (based on phase and/or amplitude measures, and measured across multiple frequency bands), dynamic metrics (for example, based on Hidden Markov Modelling), entropic measures, and spectral considerations (including periodic and aperiodic components). In addition, whilst we have incorporated all frequencies in our analyses (i.e., the full-time frequency spectra were used for our fingerprinting analyses), our choice of task necessarily means that oscillatory responses are dominated by the beta frequency band. In future comparisons of SQUIDs and OPMs, fingerprinting should be carried out both using other metrics of brain function, and other frequency bands.

Fingerprinting itself is increasingly being recognised as a useful technique as a tool to learn more about the stability of brain function in health and disease (da Silva Castanheira et al., 2021,^2024^). Nevertheless, the technique itself is still emerging and there remain methodological questions. For example, some studies undertake fingerprinting by splitting long recordings into multiple segments and correlating between segments. In such studies, whilst the data segments are statistically independent, it is not inconceivable that fingerprinting may be confounded by, for example, a systematic error in co-registration of sensor data or a unique way in which the subject was seated relative to the MEG sensors. One way to mitigate such risks is to acquire data during independent scanning sessions, but even then, it is possible that non-neural effects may contribute to identifiability—for example, in a conventional MEG system, it is possible that an individual with a small head can be differentiated from someone with a large head, based purely on signal-to-noise ratio (SNR) of the recorded signals. Here, by demonstrating that fingerprinting is possible using two MEG systems with fundamentally different architecture, different co-registration procedures, and independent (if equivalent) processing pipelines, we have added weight to the argument that the ability to carry out fingerprinting in MEG data does indeed rely on unique neural signals coming from individuals’ brains. This said, we note that cross-platform fingerprinting is not completely free of confounds; for example, fingerprinting could still relate to non-neuronal activity such as movement or blinking behaviour, if these artefacts are not completely removed either at source or by preprocessing.

In addition to fingerprinting, we demonstrated significant attentional modulation of the beta rhythm in sensory cortex. It has long been established that engaging the sensorimotor cortices leads to a drop in the amplitude of beta oscillations (Pfurtscheller & Lopes da Silva, 1999). In addition, studies have linked beta oscillations with long range connectivity within distributed networks (Brookes et al., 2011;Hipp et al., 2012;Sadaghiani et al., 2022). These findings combined have led to theories that beta oscillations exert a top-down inhibitory influence on primary cortices, which likely originates in distributed networks (seeBarone & Rossiter (2021)for a review). It is, therefore, not surprising that switching sensory attention between the left and right hands causes a change in beta amplitude. More explicitly, when an attentional cue is presented to the subject, the drop in beta amplitude in the contralateral primary sensory cortex reflects a drop in inhibition and an increase in excitability, which makes processing of sensory information in the region that maps to the attended hand more efficient. It should be made clear that this is not the first study to report this finding (Bauer et al., 2006). Further, we reported similar effects, detected using OPMs (Rivero et al., 2024) (albeit with an earlier prototype of the scanner used for the current study). However, a significant difference is that the earlier work used a more challenging realisation of the paradigm, in which more braille patterns were used. Here, we simplified the paradigm such that only two braille patterns were used. This resulted in a mean accuracy of ~97% on the task (distinct from, e.g., 51.3% in Reina Rivero et al). The fact that attentional effects remain, even in this simplified version of the task, is of interest. That the two scanner platforms were both able to detect these subtle effects provides more evidence that they measure similar information.

There are some limitations of the current study that should be mentioned. Firstly, the noise floor of our triaxial sensors is high (15 fT/√Hz) compared with both dual-axis OPMs (<10 fT/√Hz) and SQUIDs (<5 fT/√Hz). This can be offset by the improved proximity of the OPMs to the brain, which increases signal by a factor of ~4 in the cortex (Boto et al., 2017;Iivanainen et al., 2017). However, this improvement relies on sensors being placed directly on the scalp surface, whereas here we used generic rigid helmets. These come in multiple sizes which helps the array adapt to head shape and size. However, the helmets do not provide a perfect fit to all areas of the head and even small gaps cause a reduction in the amplitude of the MEG signal. Secondly, the OPM system contains only 192 channels, whereas SQUID systems contain close to 300 channels. Recent work (Hill et al., 2024) has shown that MEG SNR in source space increases linearly with the norm of the measured forward field. This, in turn, increases (nonlinearly) with channel count. This gives the SQUID system, with its higher channel density, SNR advantages relative to the OPM system. Despite its higher noise and lower channel count, our OPM-MEG instrument was still able to successfully measure both primary and attentional effects as well as carry out neural fingerprinting with a success rate similar to the SQUID device. It follows that even with SNR disadvantages, this iteration of OPM-MEG is performing well, and SNR will improve as we move to flexible helmets which adapt to head size/shape and higher channel counts, which will likely become a feature of future OPM-MEG instrumentation. Secondly, our OPM-MEG system was housed in an MSR with five layers of magnetic shielding, whereas our SQUID system was located in a three-layer MSR. This (apparently) higher requirement for shielding is, ostensibly, an argument against ultimately replacing SQUIDs with OPMs in future iterations of MEG systems. However, recent work has demonstrated the viability of three-layer shields (Holmes et al., 2022) and even two-layer shields (Holmes et al., 2025) for OPM-MEG, with the reduction in passive shielding compensated by active field cancellation. For this reason, we do not believe that shielding will ultimately prove to be a limitation. Finally, our analysis was limited to 15 individuals (60 datasets). Statistically the probabilities that we could have obtained our results by chance are extremely small (i.e., if data were completely random, it is most likely that the number of subjects correctly identified would be 0 (a probability of ~0.35) or 1 (0.4). This rapidly falls to ~0.2 for two subjects identified, ~0.05 for three subjects, and ~0.01 for four subjects). Nevertheless, it is inevitable that the addition of more subjects would increase the chances of multiple subjects having similar brain activity, and, therefore, a single subject would become less identifiable.

## 5 Conclusion

We aimed to demonstrate equivalence between an OPM and a SQUID array, when measuring MEG data during a sensory task known to generate induced oscillatory (beta-band) and evoked responses. We reasoned that, in addition to measuring primary responses (which comprise neuromagnetic fields that are stable over subjects), neural fingerprinting would prove a useful platform to test whether the subtle but repeatable differences between individuals MEG data are measurable across two independent platforms. Our results showed (1) very similar primary responses measurable using both systems; (2) both systems were able to capture attentional modulation of the beta band rhythm; and (3) fingerprinting was successful in 15 of 15 people using OPM-MEG, 14 of 15 people using SQUID-MEG, and 15 of 15 people when examining cross-platform data. Overall, results confirm that the two MEG platforms capture similar information on brain function. This finding is an important step towards proving equivalence between OPMs and SQUIDs, which, in turn, is required if OPMs are to replace SQUIDs for clinical evaluations.

## Supplementary Material

Supplementary Material

## Data Availability

All code was custom developed using MNE-Python and is available on GitHub (https://github.com/Zoe-Tanner/Demonstrating-equivalence-across-magnetoencephalography-scanner-platforms). This repository also contains a link to the data, which were acquired by authors.
