# Supplementary material for: Demonstrating equivalence across magnetoencephalography scanner platforms using neural fingerprinting
Source: Imaging Neurosci (Camb). 2025 May 21;3:IMAG.a.10. doi: 10.1162/IMAG.a.10 (PMC12319734; doi:10.1162/IMAG.a.10)
Supplement: Supplementary Material [file imag.a.10_supp.pdf]

SUPPLEMENTARY INFORMATION

Our main manuscript presents only the fingerprinting analyses applied to the induced effects, and the results shown are averaged across functional images and time-frequency spectra. Here we expand this. Figure S1A shows cross-platform fingerprinting matrices, generated by correlation of either the time-frequency spectra from sensorimotor cortices (upper panel), pseudo-T-statistical images of beta-band modulation (centre panel) and the average of the two (lower panel). Using only the time-frequency spectra, 10/15 subjects were correctly identified and using only the beta-band images, 9/15 subjects were identified. However, when combining the two metrics, all 15 were correctly identified. (Note that the lower panel is identical to the matrix presented in Figure 6).

Figure S1B shows cross platform fingerprinting applied to the evoked responses. The upper panel shows correlation of the evoked response time courses, the centre panel shows evoked response images, and the lower panel shows the two combined. Interestingly, evoked response fingerprinting was less successful with 6/15 subjects correctly identified based on evoked response time courses, 8/15 identified via evoked response images, and 10/15 based on the combined metric.

Figures S2 and S3 are equivalent to Figure S1, but show the within-platform fingerprinting performance for OPMs and SQUIDs respectively. Within-platform results also show that fingerprinting is more successful when applied to oscillatory responses compared to the evoked response. This is addressed in our discussion.

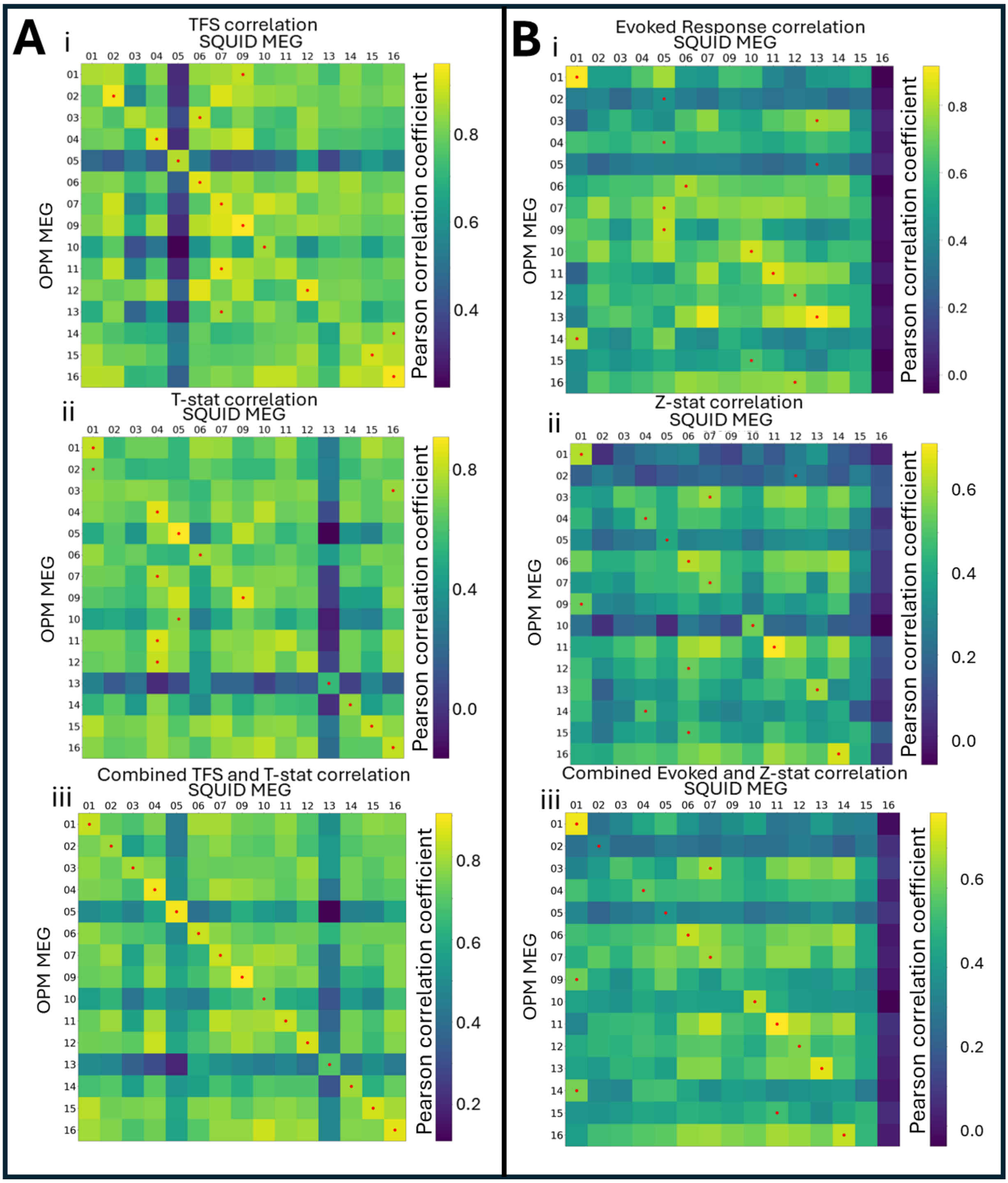

Figure S1: Cross platform fingerprinting – expanded results. *A) Fingerprinting matrices showing subject identifiability based on time-frequency spectrograms (i) pseudo-T-statistical images of beta-band modulation (ii) and the two metrics combined (iii). B) Fingerprinting matrices showing subject identifiability based on evoked response time courses (i) images of the spatial distribution of the evoked response (ii) and the two metrics combined (iii)*

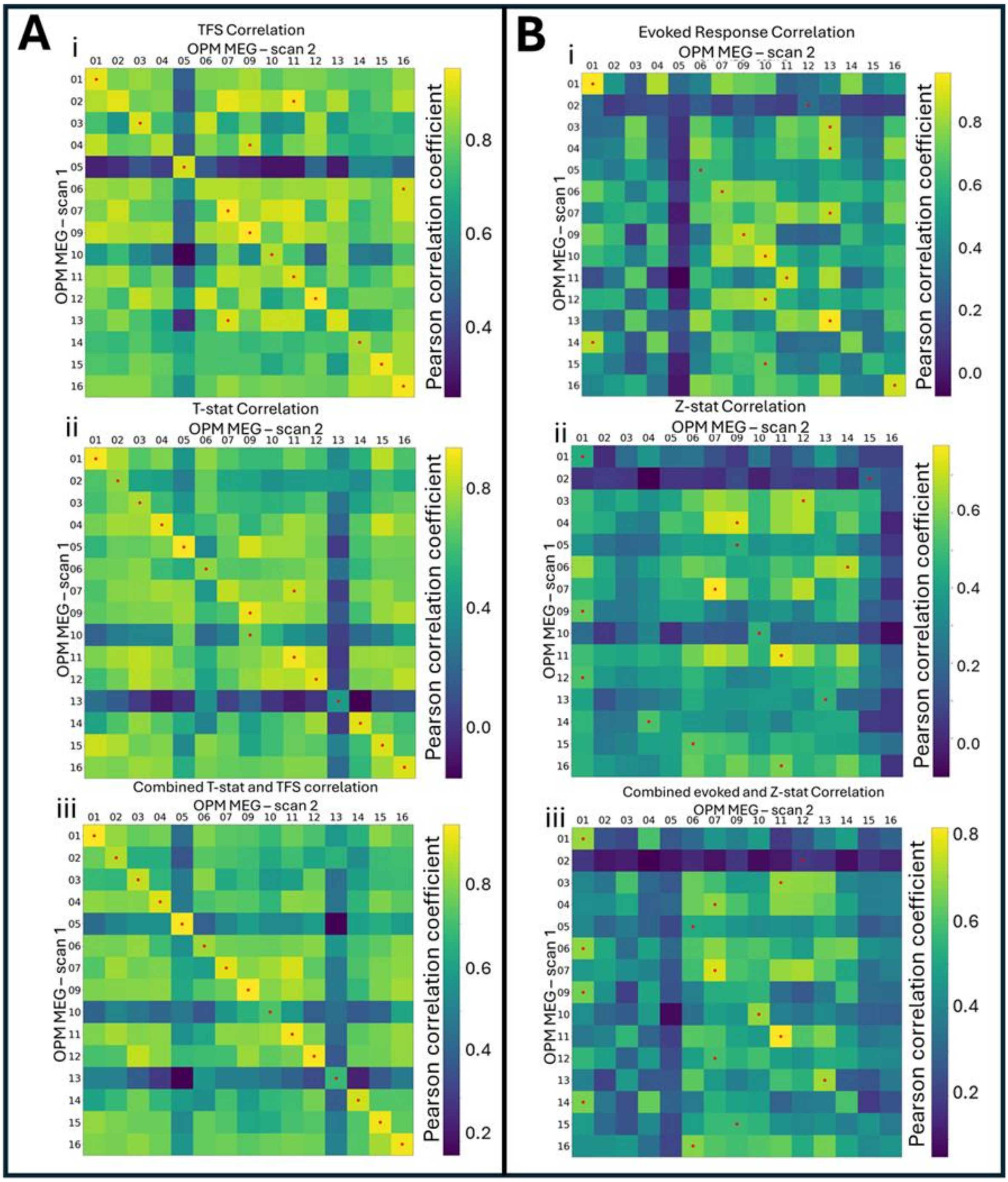

Figure S2: OPM fingerprinting – expanded results. *A) Fingerprinting matrices showing subject identifiability based on time-frequency spectrograms (i) pseudo-T-statistical images of beta-band modulation (ii) and the two metrics combined (iii). B) Fingerprinting matrices showing subject identifiability based on evoked response time courses (i) images of the spatial distribution of the evoked response (ii) and the two metrics combined (iii)*

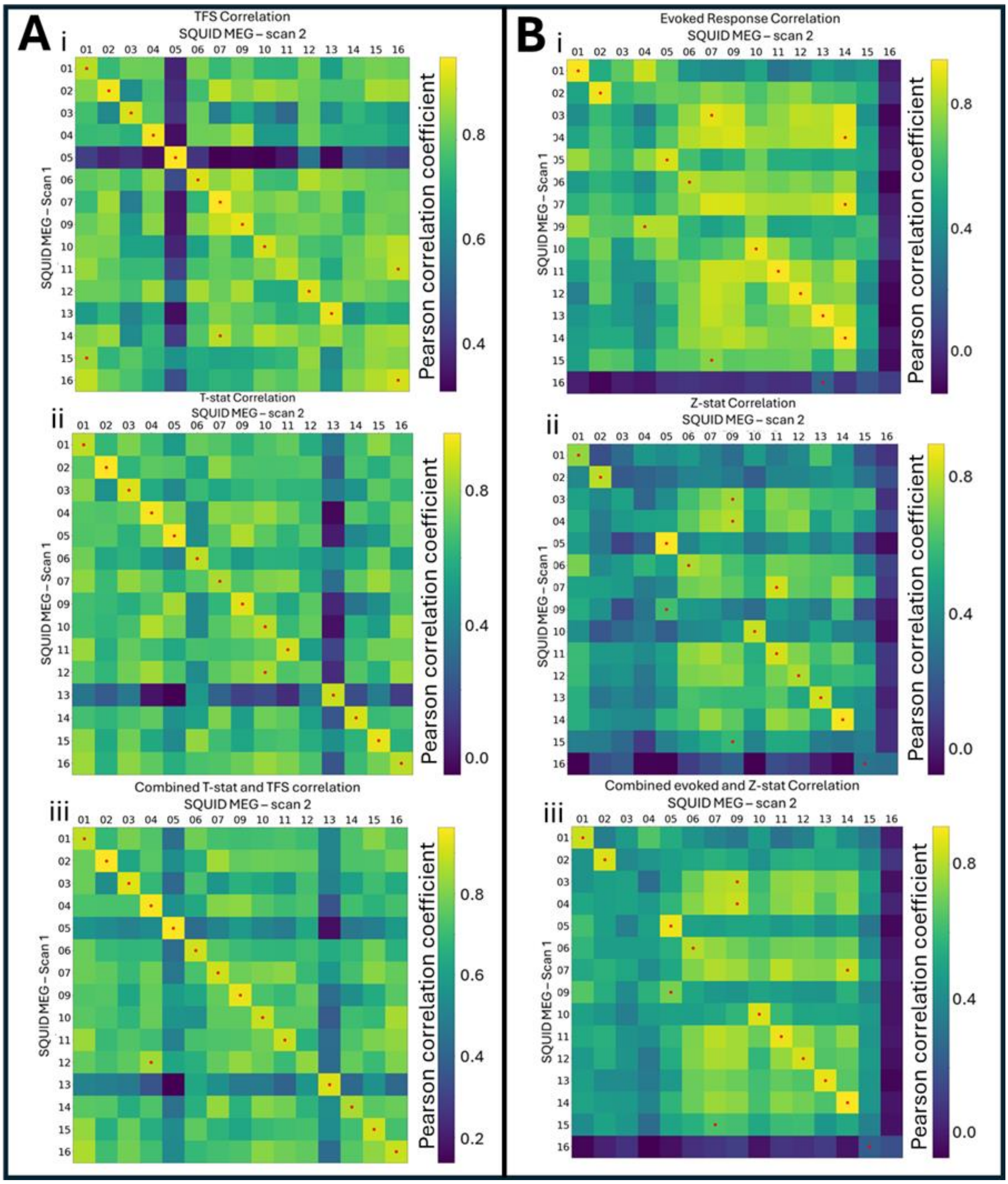

Figure S3: SQUID fingerprinting – expanded results. *A) Fingerprinting matrices showing subject identifiability based on time frequency spectrograms (i) pseudo-T-statistical images of beta-band modulation (ii) and the two metrics combined (iii). B) Fingerprinting matrices showing subject identifiability based on evoked response time courses (i) images of the spatial distribution of the evoked response (ii) and the two metrics combined (iii)*
